# Transgender Women's Voice Outcome After Laryngochondroplasty—A Systematic Literature Review

**DOI:** 10.1002/oto2.70264

**Published:** 2026-06-29

**Authors:** Eiman Abu Bandora, Hen Chaushu, Michal Icht, Narin N. Carmel Neiderman, Yotam Lior, Noa Diamant, Roni Vass, Yuval Nachalon, Yael Oestreicher‐Kedem

**Affiliations:** ^1^ Department of Otolaryngology, Head and Neck and Maxillofacial Surgery, Tel‐Aviv Sourasky University Medical Center, Gray Faculty of Medical and Health Sciences Tel Aviv University Tel Aviv Israel; ^2^ Department of Communication Disorders Ariel University Israel; ^3^ Division of Anesthesiology and Critical Care, Tel‐Aviv Sourasky University Medical Center, Gray Faculty of Medical and Health Sciences Tel Aviv University Tel Aviv Israel

**Keywords:** chondrolaryngoplasty, laryngochondroplasty, outcome, transgender, voice, women

## Abstract

**Objective:**

To systematically review the literature on voice outcomes following laryngochondroplasty (LCP).

**Data Sources:**

PubMed, Scopus, and Embase.

**Review Methods:**

A systematic review conducted in accordance with the Preferred Reporting Items for Systematic Reviews and Meta‐Analyses guidelines. Studies published in English between January 1990 and January 27, 2026, evaluating voice quality after LCP, were eligible. Included study designs were randomized controlled clinical trials, cohort studies, case‐control studies, and case series. Extracted data encompassed study design, sample size, surgical technique, voice assessment methods, and reported outcomes.

**Results:**

Of 344 articles identified, 126 duplicates were removed, and 162 articles were excluded by screening title and abstract. The full texts of the remaining 44 articles were reviewed, and 20 met the eligibility criteria, representing a cohort of 760 patients. Most studies relied on subjective or qualitative voice assessments; only two incorporated objective acoustic analyses. Postoperative voice changes were reported in 98 of 777 patients (12.6%), more in patients operated via the transcervical approach (13%), than via the transvestibular approach (5.2%). Fewer reports of voice changes were found when utilizing the transcervical technique with vocal fold localization than without (2.8% vs 29.6%, respectively). More reports of voice changes were found in studies from lead authors affiliated with Otolaryngology‐Head and Neck Surgery departments than in those affiliated with plastic surgery departments (15.3% vs 9.9%, respectively).

**Conclusions:**

Voice changes may occur following LCP, and the surgical approach may affect the risk. Larger prospective studies using standardized and objective voice measures are needed to confirm these findings.

Laryngochondroplasty (LCP), also referred to as chondrolaryngoplasty and Adam's apple reduction, is a surgical procedure aimed at reshaping the thyroid cartilage to reduce its size and anterior projection. This procedure is particularly sought after by transgender women desiring to feminize their neck appearance by altering post‐pubertal laryngeal cartilage size, shape, and projection. Feminizing gender‐affirming hormone therapy is ineffective in accomplishing cervical changes, leaving surgery as the primary option for achieving the desired neck contour.^1^, ^2^ LCP was first introduced by Wolfort et al who surgically addressed gender dysphoria related to neck appearance through a transcervical approach.^3^ Several modifications to Wolfort et al's technique were subsequently suggested with the aim of reducing the visibility of the neck scar. In 2020, the endoscopic transvestibular approach to thyroidectomy was adopted by Chung et al^4^ and by Verhasselt et al^5^ for endoscopic LCP. They reported its feasibility on cadavers, followed by Khafif et al^6^ who reported the first four cases of transgender women. Recently, a direct transvestibular approach to LCP was described.^7^, ^8^

Voice is a key gender cue and a central element of a transgender woman's identity. Vocal incongruence can cause significant distress and negatively impact various aspects of life.^9^, ^10^, ^11^ This makes voice an important consideration in the context of LCP, an elective gender‐affirming procedure designed to enhance an individual's perception of their gender identity. Despite its growing popularity, the impact of LCP on voice outcomes in transgender women remains underreported. Although LCP is intended to be voice‐sparing, postoperative voice changes can still occur. Excessive reduction of the anterior thyroid prominence may disrupt the attachment of the vocal folds to the thyroid cartilage, altering vocal fold length, tension, and height and leading to dysphonia. Over‐reduction of the thyroid ala may injure the thyroarytenoid muscle and contribute to voice changes. Direct injury to the cricothyroid muscle during LCP or postoperative local neck fibrosis can also alter tension mechanics. Additionally, intubation‐related trauma to the vocal folds may occur.

Given the central role of voice in gender dysphoria, the potential risk of postoperative voice changes following LCP, and the scarcity of objective data in the literature, this study aims to systematically review the available evidence on voice outcomes after LCP.

## Methods

### Search Strategy

A systematic literature review was conducted according to the Preferred Reporting Items.

For Systematic Reviews and Meta‐Analyses (PRISMA) guidelines.^12^ A comprehensive search through multiple databases including MEDLINE (PubMed), Scopus and Embase was performed to identify articles published in English addressing voice quality following LCP in transgender women between January 1990 and January 27, 2026. The following search key terms were used in combinations (“tracheal shave” OR “chondrolaryngoplasty” OR “laryngochondroplasty” OR “thyroid cartilage reduction”) AND (“male‐to‐female” OR “transgender”). Reference lists of relevant studies were also reviewed. All references were uploaded to Covidence (Veritas Health Innovation Ltd., Melbourne, Australia) and duplicates were removed.

### Study Collection

Inclusion criteria according to the study design were any randomized clinical trials, prospective and retrospective cohorts, case‐control designs, and case series that addressed voice quality following LCP, using either objective or subjective measures. Exclusion criteria included articles in languages other than English, conducted with patients under 18 years old, or those lacking a voice outcome. Two authors (EAB and YOK) independently screened the titles and abstracts for relevance, followed by full‐text review of the relevant studies. Any disagreements were resolved through discussion.

### Data Extraction

Data were extracted systematically, including author, study design, number of patients, surgical technique, voice assessment methods and reported voice outcomes. Variables were recorded in Microsoft Excel (Microsoft Corporation).

### Risk of Bias Assessment

Risk of bias was independently assessed by 2 coauthors using the Newcastle–Ottawa Quality Assessment Scale for nonrandomized cohort studies and the Joanna Briggs Institute (JBI) critical appraisal checklist for case reports.^13^, ^14^

## Results

### Literature Search

**Figure 1:**
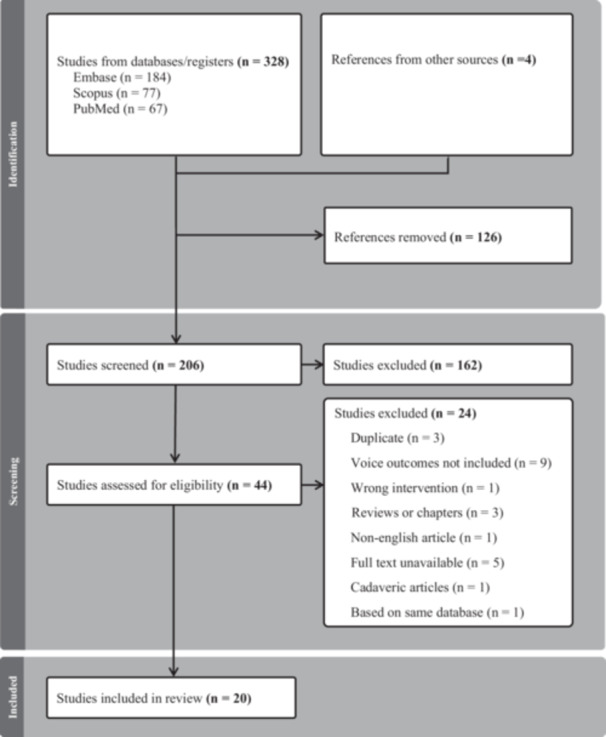
Process of identification, inclusion and exclusion of studies for the literature review.

The review identified 344 studies (Figure 1). After removing duplicates, 206 titles and abstracts were screened. Of those screened, 44 underwent full‐text review, 20 of which were found to report voice outcomes following LCP (Table 1).^6^, ^8^, ^15^, ^16^, ^17^, ^18^, ^19^, ^20^, ^21^, ^22^, ^23^, ^24^, ^25^, ^26^, ^27^, ^28^, ^29^, ^30^, ^31^, ^32^ Reasons for exclusion are detailed in Figure 1. Risk of bias assessment is presented in Supplements 1 and 2.

**Table 1:** Voice Outcomes Following Laryngochondroplasty According to Surgical Approach.^6^, ^8^, ^15^, ^16^, ^17^, ^18^, ^19^, ^20^, ^21^, ^22^, ^23^, ^24^, ^25^, ^26^, ^27^, ^28^, ^29^, ^30^, ^31^, ^32^

Surgical approach	First author, publication year ref	Specialty of the leading author	Voice outcome measure	Postoperative voice change (Number of patients with voice change/all study patients)	Duration of voice change (Duration specified in article)
Transcervical without vocal fold localization	Wolfort FG, 1990^23^	P	Subjective patient report	Y (21/31)	Transient (Up to 6 months)
	Al Jassim A, 2006^19^	O	Subjective patient report	Y (1/1)	Transient (48 hours)
	Aries MM, 2020^24^	O	F0, GRABS scale	N (0/15)	
	Zeng Y, 2023^21^	P	Subjective patient report Physician observation	Y (2/34)	Transient (3 months)
Transcervical with vocal fold localization	Conrad K, 2003^25^	P	Subjective patient report	N (0/10)	
	Spiegel JH, 2008^26^	O	Subjective patient report	N (0/31)	
	Cohen MB, 2018^20^	O	CAI	Y (5/45)	Transient (NS)
	Tang CG, 2020^27^	O	Subjective patient report	N (0/91)	
Transcervical, unknown surgical method	Strickland L, 2022^15^	O	Subjective patient report	Y (1/1)	Long term (21 months)
	Nuyen B, 2023^16^	O	Subjective patient report Stroboscopy	Y (27/94)	Long term (Average 4.9 years)
	Khafif A 2020^6^	O	Stroboscopy, vocal range	N (0/4)	
	David AP, 2022^8^	P	CAI	N (0/6)	
	Eggerstedt M, 2022^28^	P	Subjective patient report	N (0/77)	
Transvestibular	Shoffel‐Havakuk H, 2023^29^	O	Subjective patient report Stroboscopy	N (0/12)	
	Oestriecher‐Kedem Y, 2024^30^	O	Subjective patient report	Y (8/20)	NS
	Haddad R, 2025^31^	O	Physician observation	N (0/15)	
Transcervical and transvestibular	Deng IY, 2025^22^	P	Not specified	Y (17/246)	Transient (NS)
	Kondamuri N,2023^17^	O	Subjective patient report	Y (1/8)	Long term (NS)
Not specified	Hughes C,2024^18^	SLP	Subjective patient report Acoustic analysis	Y (1/1)	Long term (7months)
	Jahnavi, 2025^32^	O	Subjective patient report Stroboscopy	Y (14/35)	NS

Abbreviations: CAI, chondrolaryngoplasty aesthetic instrument; GRABS, grade, roughness, asthenia, breathiness, strain; N, no; NS, not specified; O, otolaryngology, head and neck surgery; P, plastic surgery; SLP, speech and language pathology; Y, yes.

### Study Characteristics and Patient Demographics

Twenty eligible studies, comprising 777 patients were included (Table 2). Thirteen studies were retrospective,^8^, ^15^, ^18^, ^19^, ^20^, ^21^, ^22^, ^23^, ^26^, ^27^, ^28^, ^30^, ^32^ 6 were prospective,^6^, ^16^, ^17^, ^24^, ^25^, ^29^ and 1 study did not specify its design.^31^ Sample sizes ranged from single case reports to a large cohort of 246 patients.^22^ Most studies were conducted in North America (USA: 13 studies; Canada: 1), followed by Europe (UK: 2, France: 1), Asia (China: 1, Israel: 3), and South America (Brazil: 1). Patient age (mean or median) was reported in 12 studies, ranging from 21 to 41 years.^6^, ^16^, ^17^, ^21^, ^22^, ^24^, ^26^, ^27^, ^28^, ^29^, ^30^, ^31^

**Table 2:** Studies Included In the Literature Review and Their Cohorts’ Characteristics

First author, publication year ref	Study type	Country	Gender (Number of patients)	Mean age in years (Range)	Surgical approach	Concomitant surgical interventions in some patients	Mean follow‐up time in months (Range)
Wolfort FG, 1990^23^	R	USA	TW (20) CM (11)	NA	TC	‐	120 (4‐204)
Conrad K, 2003^25^	P	Canada	TW (8) CM (2)	NA	TC	‐	24 (NA)
Al Jassim A, 2006^19^	R	UK	TW (31)	NA	TC	NA	NA
Spiegel JH, 2008^26^	R	USA	NA (31)	41	TC	‐	NA
Cohen MB, 2018^20^	R	USA	NA (45)	NA	TC	NA	NA
Tang CG, 2020^27^	R	USA	TW (79)	31.4	TC	NA	20.7 (NA)
Strickland L, 2022^15^	R	USA	TW (1)	30	TC	‐	NA
Aries MM, 2020^24^	P	Brazil	TW (11)	31.7	TC	‐	15.3 (6‐25)
Khafif A, 2020^6^	P	Israel	TW (4)	NA	Endoscopic TV	‐	2
David AP, 2022^8^	R	USA	NA (6)	NA	Endoscopic TV	‐	6.62 (NA)
Eggerstedt M, 2022^28^	R	USA	TW (77)	27	Direct TV	Glottoplasty Facial feminization Genioplasty	8.74 (1‐30)
Nuyen B, 2023^16^	P	USA	TW (94)	36.7	TC	NA	NA
Shoffel‐Havakuk H, 2023^29^	P	Israel	TW (10) CW (1) CM (1)	26.7	Endoscopic TV	‐	2
Zeng Y, 2023^21^	R	China	TW (4) CW (29) CM (1)	27.12	TC	NA	NA (6‐60)
Kondamuri N, 2023^17^	R	USA	NA (8)	NA	NA	Glottoplasty	NA
Hughes C, 2024^18^	P	UK	TW (1)	25	NA	Cranioplasty Rhinoplasty Genioplasty	NA (3‐7)
Oestriecher‐Kedem Y, 2025^30^	R	Israel	TW (20)	28	Direct TV	Genioplasty Gonioplasty	11 (1.5‐25.5)
Haddad R, 2025^31^	NA	France	TW (15)	28.7	Endoscopic TV TC	Genioplasty Gonioplasty	NA
Deng I, 2025^22^	R	USA	NA (246)	30.7	Endoscopic TV TC	NA	NA (0.3‐48.5)
Jahnavi, 2025^32^	R	USA	TG (35)	NA	NA	NA	NA

Abbreviations: CM, cisgender man; CW, cisgender woman; NA, not addressed; P, prospective; R, retrospective; TC, transcervical; TV, transvestibular; TW, transgender woman.

### Surgical Indication and Approach

Gender‐affirming care was the primary indication for LCP across most studies, although some cohorts also included cisgender men and women (Table 2).^21^, ^25^, ^29^ The transcervical approach was most frequently used (n = 10).^15^, ^16^, ^19^, ^20^, ^21^, ^23^, ^24^, ^25^, ^26^, ^27^ Four studies employed an endoscopic transvestibular approach ^6^, ^8^, ^29^, ^31^ and 2 described a direct transvestibular approach.^28^, ^30^ One study reported both transcervical and endoscopic transvestibular approaches,^22^ while three studies did not specify the surgical approach.^17^, ^18^, ^32^ Several studies also reported concurrent facial feminization procedures,^17^, ^18^, ^28^, ^30^, ^31^ reflecting a broader trend toward comprehensive gender‐affirming surgery. Postoperative follow‐up, reported in 12 studies, ranged from 7 days to 17 years.

Considerable heterogeneity was observed in study design, surgical approach, and follow‐up duration.^6^, ^8^, ^17^, ^21^, ^22^, ^23^, ^24^, ^25^, ^27^, ^28^, ^29^, ^30^, ^32^

### Voice Outcomes

Voice outcomes following LCP were assessed using both qualitative and quantitative methods (Table 1, Supplement S3, available online). Most studies (n = 17) relied on qualitative evaluations such as subjective patient‐reported outcomes or physician‐perceived changes.^8^, ^15^, ^16^, ^18^, ^19^, ^20^, ^21^, ^22^, ^23^, ^25^, ^26^, ^27^, ^28^, ^29^, ^30^, ^31^ A smaller incorporated stroboscopic examination (n = 4)^6^, ^16^, ^29^ or standardized tools such as the Chondrolaryngoplasty Aesthetic Instrument (CAI) and the GRBAS scale.^8^, ^20^, ^24^ Quantitative acoustic analyses were infrequently reported, with only two studies reporting objective voice acoustic analyses.^17^, ^24^

Objective acoustic outcomes were reported in 2 studies.^17^, ^24^ Aires et al measured mean fundamental frequency preoperatively and at 1 month postoperatively, finding no significant differences.^24^ Hughes et al analyzed the speech and singing voice of one transgender singer before and after facial feminization with LCP.^18^ They observed transient, statistically significant reductions in the difference in amplitude between the first and second harmonics (H1‐H2) and in the difference in amplitude between the first harmonic and the amplitude of the strongest harmonic within the first formant (H1‐A1) at 3 months postoperatively, with partial return to baseline by 7 months. Because higher H1 to H2 values are associated with increased breathiness or hoarseness, these reductions were considered unlikely to result solely from surgical effects and may have reflected behavioral factors such as vocal fatigue, artistic choice, or deliberate modification of vocal quality postoperatively.

Eleven of 20 studies reported some degree of voice change, affecting 98 of 777 patients (12.6%).^15^, ^16^, ^17^, ^18^, ^19^, ^20^, ^21^, ^22^, ^23^, ^30^ The duration of these voice changes could not be clearly determined. Five studies, including a total of 357 patients, reported “transient” voice changes in 46 patients (12.9%).^19^, ^20^, ^21^, ^22^, ^23^ In 3 of these 5 studies, transient changes were reported to last between 48 hours and 6 months.^19^, ^21^, ^23^ Four studies, including a total of 104 patients, reported “long‐term” voice changes in 30 patients (28.8%).^15^, ^16^, ^17^, ^18^ In 3 of these 4 studies, long‐term changes were reported over time frames ranging from 7 to 59 months.^16^, ^17^, ^18^ Two studies did not specify whether the voice changes were transient or long‐term.^30^, ^32^

Only one study directly compared LCP surgical approaches, finding fewer voice changes with endoscopic transvestibular versus transcervical LCP [1/39 (2.6%) vs 16/207 (7.7%), respectively].^22^ Across all 20 studies, voice changes occurred in 73 of 560 patients (13%) treated via the transcervical approach, compared with 9 of 173 (5.2%) treated via the transvestibular approaches (endoscopic or direct). When comparing voice outcomes by type of transcervical technique, fewer reports of voice changes were found with the “with vocal fold localization” method than with the “without vocal fold localization” method [5/177 (2.8%) *vs* 24/81 (29.6%), respectively] (Table 1). When comparing voice outcomes according to the departmental affiliation of the lead author, fewer reports of voice changes were seen in studies from plastic surgery departments than in those from Otolaryngology–Head and Neck Surgery departments [40/404 (9.9%) vs 57/372 (15.3%), respectively] (Table 1).

## Discussion

Both physical appearance and voice quality are critical components of gender identity and central to the social integration of transgender women.^33^ While the cosmetic benefits of LCP are well established, its impact on voice, a fundamental gender cue and an essential element of identity, has been inconsistently reported. This review is the first to synthesize the available evidence on voice outcomes following LCP.

Considerable heterogeneity was observed in voice outcome assessment. Most studies (n = 16) relied on subjective measures such as patient‐reported outcomes or physician perception.^8^, ^15^, ^16^, ^17^, ^18^, ^19^, ^21^, ^23^, ^25^, ^26^, ^27^, ^28^, ^29^, ^30^, ^31^, ^32^ Only a limited number used standardized tools, including the GRBAS scale, the CAI and objective acoustic analyses.^8^, ^17^, ^20^, ^24^ Hughes et al, using quantitative acoustic measures, reported statistically significant, but transient, changes in formant frequencies, suggesting temporary alterations in vocal control in one patient.^18^ In contrast, Aires et al, did not find significant changes in fundamental frequency postoperatively.^24^ These methodological inconsistencies limit definitive conclusions and underscore the need for standardized, objective voice assessment tools. Future prospective studies comparing surgical approaches with uniform voice outcome protocols are particularly warranted.

Across the 20 included studies, involving 777 patients, voice changes were reported in 98 patients (12.6%), about half of which were transient.^15^, ^16^, ^17^, ^18^, ^19^, ^20^, ^21^, ^22^, ^23^, ^30^ Although the definitions of “transient” and “long‐term” voice changes varied across studies, existing data suggest that a meaningful subset of patients experience voice alterations beyond the immediate postoperative period. These findings highlight both the clinical relevance of voice outcomes after LCP and the current limitations in standardized reporting and follow‐up. Given this clinically meaningful risk of vocal fold injury associated with LCP, the possibility of postoperative voice changes should be explicitly discussed with patients as part of the informed consent process.

Several mechanisms have been proposed to explain the observed changes. Disruption of the anterior commissure has been implicated as a key anatomical factor. Nuyen et al, reported persistent hoarseness, rough vocal quality, and pitch lowering in 27 of 94 patients, attributing these outcomes to anterior commissure disruption confirmed by bedside needle localization under flexible laryngoscopy.^16^ Jahnavi and Thomas observed vocal fold detachment during feminization laryngoplasty following LCP in 29% of patients (n = 35).^32^

To minimize the risk of vocal fold injury and preserve the anterior commissure, several surgical refinements have been suggested. Spiegel et al, described the use of external translaryngeal needle insertion under general anesthesia, guided by fiberoptic bronchoscopy and a laryngeal mask airway, to accurately localize and protect the anterior commissure.^26^ Zeng et al modified the LCP technique by incorporating bilateral platysma flap advancement to camouflage the thyroid notch and reduce tissue tension, potentially optimizing both vocal and aesthetic outcomes.^21^ The substantially lower rate of reported voice changes observed with the “vocal fold localization” transcervical technique compared to “without vocal fold localization” (2.8% vs 29.6%, respectively) supports the routine use of this method to better preserve voice outcomes following transcervical LCP.

In addition, postoperative behavioral adaptations may contribute to the postoperative voice change. Hughes et al, evaluated both speech and singing voice in a transgender singer following LCP and suggested that postoperative voice outcomes may be influenced not only by anatomical alterations but also by behavioral and social factors.^18^ Collectively, these findings underscore the multifactorial nature of voice outcomes following LCP.

Surgical approach may also play a role. Only one study directly compared voice outcomes by surgical approach, reporting a lower incidence of postoperative voice changes among patients treated via the transvestibvular approach (2.6%) compared with those treated via the transcervical approach (7.7%).^22^ Consistent with this, our pooled analysis also demonstrated a lower rate of reported postoperative vocal changes among patients undergoing the transvestibular versus the transcervical approach (5.2% vs 15.5%, respectively). While these findings suggest a potentially lower risk of voice change with the transvestibular approach, substantial heterogeneity in outcome assessment and reporting across centers limits the ability to draw definitive conclusions.

We observed a higher reported rate of voice changes in studies from Otolaryngology Head and Neck Surgery departments compared with those from Plastic Surgery departments (15.3% vs 9.9%, respectively). Several factors may explain this difference, which likely reflect variations in clinical focus, outcome assessment, and reporting practices rather than true differences in surgical safety. Voice evaluation is central to otolaryngologic practice, and otolaryngologists, especially those with laryngology expertise, are more likely to use detailed, multidimensional voice assessments and to detect and report even subtle changes in voice quality, pitch, or function. In contrast, plastic surgery studies often emphasize cosmetic outcomes and patient satisfaction with neck contour and may be less likely to systematically assess or report minor or transient voice changes. Additionally, referral and selection biases may contribute, as patients experiencing postoperative voice issues are more likely to present to otolaryngology clinics and be included in studies from those departments.^16^, ^32^

Our review is limited by several methodological weaknesses. Significant heterogeneity exists in study designs, surgical approaches, outcome definitions, and follow‐up durations. Most included studies were retrospective, had small sample sizes, and lacked control groups. Furthermore, few studies distinguished the specific impact of LCP on voice from other concurrent gender‐affirming interventions, such as glottoplasty or voice therapy, which may independently influence vocal outcomes. The predominant reliance on qualitative and subjective measures further constrains the generalizability and reproducibility of findings.

## Conclusions

While most patients do not experience lasting or significant vocal impairment following LCP, voice changes may occur postoperatively and should be discussed as part of preoperative counseling. The surgical approach may affect voice outcome. Given the critical importance of voice in gender affirmation, future prospective studies using standardized, objective, and validated voice outcome measures are essential.

## Author Contributions

**Eiman Abu Bandora**, writing—original draft preparation (lead), data curation (lead), investigation (lead); **Hen Chaushu**, writing—original draft preparation (supporting), conceptualization (supporting); **Michal Icht**, writing—original draft preparation (supporting), conceptualization (supporting); **Narin N. Carmel Neiderman**, conceptualization (supporting), methodology (supporting); **Yotam Lior**, conceptualization (supporting), review (supporting); **Noa Diamant**, writing—review (supporting); **Yuval Nachalon**, Writing—review (supporting); **Roni Vass**, writing—review (supporting); **Yael Oestreicher‐Kedem**, conceptualization (lead), original draft preparation (supporting), writing—review and editing (lead).

## Disclosures

The study was presented at the Cutting‐Edge Laryngology 2024 meeting, London, United Kingdom, 2‐4 October, 2024, and at the Israeli Otolaryngology Head and Neck Surgery Association Annual Meeting, Tel Aviv, Israel, 19‐20 March, 2025.

### Competing interests

None.

### Funding source

None.

## Supporting information


**Supplement 1:** Risk of bias assessment using the Newcastle‐Ottawa Quality Assessment Scale Criteria.

**Supplement 2:** Risk of bias assessment using the Joanna Briggs Institute Critical Appraisal Checklist for Case Reports.

**Supplement 3:** Nature of Voice Change Following Laryngochondroplasty.
