# Supplementary material for: Transgender Women's Voice Outcome After Laryngochondroplasty—A Systematic Literature Review
Source: OTO Open. 2026 Jun 29;10(3):e70264. doi: 10.1002/oto2.70264 (PMC13313092; doi:10.1002/oto2.70264)
Supplement: Supplementary file 1 — Supplement 1: Risk of bias assessment using the Newcastle‐Ottawa Quality Assessment Scale Criteria. [file OTO2-10-e70264-s001.docx]

Supplement 1

| First Author, Publication Year ^ref^ | Representativeness of the exposed cohort | Selection of the non-exposed cohort | Ascertainment of exposure | Demonstration that outcome of interest was not present at start of study | Comparability of cohorts based on the design or analysis controlled for confounders | Assessment of outcome | Follow-up long enough for final outcome assessment | Adequacy of follow-up of cohorts | Total score |
| --- | --- | --- | --- | --- | --- | --- | --- | --- | --- |
| Wolfort FG, 1990 ^16^ | 1 | 0 | 1 | 0 | 0 | 0 | 1 | 1 | 4 |
| Conrad K, 2003 ^17^ | 1 | 0 | 1 | 0 | 0 | 0 | 0 | 1 | 3 |
| Spiegel JH, 2008 ^19^ | 1 | 0 | 1 | 0 | 0 | 0 | 0 | 1 | 3 |
| Cohen MB, 2018 ^20^ | 1 | 0 | 1 | 0 | 0 | 0 | 0 | 0 | 2 |
| Tang CG, 2020 ^21^ | 1 | 0 | 1 | 0 | 0 | 0 | 0 | 0 | 3 |
| Aries MM, 2020 ^23^ | 1 | 0 | 1 | 1 | 0 | 1 | 1 | 0 | 5 |
| Khafif A, 2020 ^6^ | 1 | 0 | 1 | 1 | 0 | 1 | 0 | 1 | 5 |
| David AP, 2022 ^8^ | 1 | 0 | 0 | 0 | 0 | 0 | 1 | 1 | 3 |
| Eggerstedt M, 2022 ^24^ | 1 | 0 | 1 | 0 | 0 | 0 | 1 | 1 | 4 |
| Nuyen B, 2023 ^25^ | 1 | 0 | 1 | 1 | 0 | 1 | 1 | 1 | 6 |
| Shoffel-Havakuk H, 2023 ^25^ | 1 | 0 | 1 | 1 | 0 | 0 | 0 | 1 | 4 |
| Zeng Y, 2023 ^26^ | 1 | 0 | 1 | 0 | 0 | 0 | 0 | 1 | 3 |
| Kondamuri N, 2023 ^27^ | 1 | 1 | 1 | 0 | 0 | 0 | 0 | 0 | 3 |
| Oestriecher-Kedem Y, 2024 ^29^ | 1 | 0 | 1 | 0 | 0 | 0 | 1 | 1 | 4 |
| Haddad R, 2025 ^30^ | 1 | 0 | 1 | 0 | 0 | 0 | 0 | 1 | 3 |
| Deng I, 2025 ^31^ | 1 | 0 | 1 | 0 | 0 | 0 | 1 | 0 | 3 |
| Jahnavi, 2025^32^ | 1 | 0 | 1 | 0 | 0 | 1 | 0 | 1 | 4 |

1-low bias risk for the respective category; 0 - high bias risk for the respective category

Total risk of bias grade: Very high (0 to 3 points); high (4 to 6 points); low (7 to 9 points)
