# Supplementary material for: Transgender Women's Voice Outcome After Laryngochondroplasty—A Systematic Literature Review
Source: OTO Open. 2026 Jun 29;10(3):e70264. doi: 10.1002/oto2.70264 (PMC13313092; doi:10.1002/oto2.70264)
Supplement: Supplementary file 2 — Supplement 2: Risk of bias assessment using the Joanna Briggs Institute Critical Appraisal Checklist for Case Reports. [file OTO2-10-e70264-s002.docx]

Supplement 2

| First Author, Publication Year ^ref^ | 1 | 2 | 3 | 4 | 5 | 6 | 7 | 8 | Total score |
| --- | --- | --- | --- | --- | --- | --- | --- | --- | --- |
| Al Jassim A, 2006 ^18^ | Y | Y | Y | Y | Y | Y | N | Y | 7 |
| Strickland L, 2022 ^22^ | Y | Y | Y | Y | Y | U | U | Y | 6 |
| Hughes C, 2024 ^28^ | Y | Y | Y | Y | Y | Y | Y | Y | 8 |
| Y-yes; N-no,U-unclear | | | | | | | | | |

1. Were patient’s demographic characteristics clearly described?
2. Was the patient’s history clearly described and presented as a timeline?
3. Was the current clinical condition of the patient on presentation clearly described?
4. Were diagnostic tests or assessment methods and the results clearly described?
5. Was the intervention(s) or treatment procedure(s) clearly described?
6. Was the post-intervention clinical condition clearly described?
7. Were adverse events (harms) or unanticipated events identified and described?
8. Does the case report provide takeaway lessons?

Risk of bias summary for all studies assessed with the JBI Critical Appraisal Tool. “Yes,” “No,” and “Unclear” are responses to the questions listed in the bottom half of the figure for each study. The overall score is the sum of the number of “Yes” responses.
