# Supplementary material for: Transgender Women's Voice Outcome After Laryngochondroplasty—A Systematic Literature Review
Source: OTO Open. 2026 Jun 29;10(3):e70264. doi: 10.1002/oto2.70264 (PMC13313092; doi:10.1002/oto2.70264)
Supplement: Supplementary file 3 — Supplement 3: Nature of Voice Change Following Laryngochondroplasty. [file OTO2-10-e70264-s003.docx]

Supplement 3: Nature of Voice Change Following Laryngochondroplasty

| First Author, Publication Year ^ref^ | Number of patients with voice change/all study patients | Nature of Voice Change |
| --- | --- | --- |
| Wolfort FG, 1990 ^15^ | 21/31 | Hoarseness, weak voice |
| Al Jassim A, 2006 ^17^ | 1/1 | Weak voice |
| Cohen MB, 2018 ^19^ | 5/45 | Somewhat to moderately different voice |
| Strickland L, 2022 ^21^ | 1/1 | Hoarseness, voice breaks, lowered pitch, and difficulty singing. |
| Nuyen B, 2023 ^24^ | 27/94 | Deepened speaking pitch (18/27), loss of upper register (5/27), rough voice (6/27) |
| Zeng Y, 2023^26^ | 2/34 | Hoarseness |
| Oestriecher-Kedem Y, 2025 ^31^ | 8/20 | More feminine voice (n=2), reduced vocal range(n=4), a less throaty voice (n=1), vocal fatigue (n=1) |
| Deng I, 2025 ^30^ | 17/246 | NS |
| Kondamuri N,2023 ^27^ | 1/8 | NS |
| Hughes C,2024 ^28^ | 1/1 | Altered speech and singing parameters. For speech, H1-H2 and H1-A1 were significantly lower at 3 and 7 months postoperatively. F1 decreased after 3 months.  For singing, both H1-H2 and H1-A1 were significantly lower at 3 months postoperatively. H1-H2 increased the 7 months postoperative recording compared with the 3 months postoperative recording. F1 increased and F3 decreased postoperatively. Temporary changes in voice control. |
| Jahnavi, 2025^32^ | 14/35 | Limited upper vocal range. Asymmetric vocal fold tension on phonation on stroboscopy (n=10) and vocal fold detachment spotted during feminization laryngoplasty. |
